# Precision Physical Distancing for COVID-19: An Important Tool in Unlocking the Lockdown

**DOI:** 10.4269/ajtmh.20-0359

**Published:** 2020-05-18

**Authors:** Daniel G. Bausch

**Affiliations:** 1 UK Public Health Rapid Support Team, Public Health England/London School of Hygiene & Tropical Medicine, London, United Kingdom;; 2 Department of Disease Control, Faculty of Infectious and Tropical Diseases, London School of Hygiene & Tropical Medicine, London, United Kingdom

## Abstract

In response to the COVID-19 pandemic, in addition to the more routine public health measures, many countries have implemented “lockdowns”—closing borders, restricting international travel, and placing severe limitations on individual movement and group gatherings. While lockdowns may be an important tool to limit transmission, they come at a potentially great cost with regard to economic impact, mental health consequences, and increased morbidity and mortality from non–COVID-19 diseases. Furthermore, implementation of the required draconian measures may be difficult in some settings because of logistical, economic, and sociocultural impediments, especially in many low- and middle-income countries. Governments and health authorities must chart a course on how to “unlock” or control transmission where lockdowns are not feasible. “Precision physical distancing”—distancing tailored and optimized to specific physical, social, cultural, political, and economic contexts and to specific groups and settings—is proposed and discussed here as an important tool in the control of COVID-19. It has the advantages of being low cost, adaptable to diverse sociocultural and economic settings through community ownership and local action, and more easily monitored and potentially enforced than less precise measures. Precision physical distancing can be one important component of a sustainable long-term solution that is proportionate to the risk yet does not have a disproportionate impact on society and the economy, allowing a partial return to normal activities, with the community as an essential partner.

The world is presently engulfed in the pandemic of COVID-19, with millions of cases and hundreds of thousands of deaths worldwide. In response to the crisis, many countries have implemented “lockdowns”—closing borders, restricting international travel, and placing severe limitations on individual movement and group gatherings. In Wuhan, China, where COVID-19 is thought to have first emerged, all journeys in and out of the city were banned; public transport suspended; private cars barred from roads; businesses, schools, and universities closed; and severe limits placed on individual mobility outside of residences. As the outbreak there worsened, authorities ordered house searches for potentially infected individuals, who were then forced into quarantine. As the pandemic has spread, countries around the world have also implemented various forms of lockdown, although few with as stringent restrictions as China.

Although the lockdown in China is credited with stemming COVID-19 transmission, the capacity to effectively implement strict lockdowns elsewhere is far from certain.^1^ Such draconian measures may be especially difficult in countries and cultures in which individual liberties and freedom of movement are taken as political and social rights perceived to outweigh personal sacrifice for a common good. Few countries have the state-run systems, technological surveillance capacity, and legal framework to enforce the strict measures implemented in China.

Although a strict lockdown may intuitively be the most efficacious measure to limit transmission, it comes at a potentially great cost with regard to economic impact, mental health consequences, and increased morbidity and mortality from non–COVID-19 diseases. China’s GDP may fall by 9% in the first quarter of 2020 compared with the same period in 2019.^2^ Although large economic aid packages being implemented by numerous governments may ease economic burdens, these will likely be too slow to save many small business owners and their employees.

Regardless of whether a lockdown can be successfully initiated, in most countries and cultures, it is certainly not sustainable for the long term. Stringent lockdowns and working from home are unlikely to be implementable in many low- and middle-income countries, where a significant proportion of the population depends on performing physical labor with daily pay in a cash economy, and where indoor plumbing, running water, electricity, and internet access are not givens. Under India’s strict lockdown, millions struggle for food.^3^ A Malawi high court and political leaders in some other countries have suspended lockdowns because of lack of adequate provision for the poor. In these circumstances, sheltering at home may not only not be possible but may also even enhance the risk of transmission in often overcrowded households. Informal urban settlements, refugee camps, and camps for internally displaced persons in some developing countries present a still greater challenge.^4,5^

In the worst-case scenarios, lockdowns may result in civil unrest, as has been seen in Nigeria, a country where 48% of the population (96 million people) live in extreme poverty. Although there is a stable supply of food in resource-rich nations, even there, lockdowns, especially if prolonged, may eventually lead to food insecurity and a restless population, with the potential for protests and civil unrest. Imposition of a stringent lockdown in Jordan met with chaos and over a thousand arrests when access to food was threatened, forcing King Abdullah II to call in security forces and, at least temporarily, lessen restrictions.^6^ In addition to the physical and social disruption civil unrest brings, if large masses accrue in close contact to protest in the streets, then the rationale for and benefit of the lockdown are ironically negated.

Given the many challenges and uncertainties, government authorities around the world are agonizing over how and whether to implement lockdowns, and for how long. These decisions are often guided by mathematical models of transmission, which may be limited in their accuracy, especially when reliable health and demographic data are unavailable. To add to the challenge, most experts anticipate multiple waves of COVID-19 transmission, meaning that a strategy of strict lockdown that might be necessary and tolerated for a short period is not a viable long-term strategy. Recognizing the long-term nature of the fight, WHO Director General Dr. Tedros Adhanom Ghebreyesus recently stated that the world needed “to live with this virus until we can develop a vaccine to get rid of it.”^7^ Regardless of whether lockdowns are feasible to begin with or sustainable for the long term, governments and health authorities must chart a course on how to “unlock.”

One fundamental and widely accepted approach to “living with the virus” is social distancing. Many public health specialists prefer the term “physical distancing” to emphasize the need for continued social interaction and support, albeit without physical interaction. Guidelines for physical distancing generally entail remaining out of congregate settings, avoiding mass gatherings, and maintaining a safe distance from others to limit the ability of a pathogen to spread. Although virtually all health authorities recommend physical distancing in response to the COVID-19 pandemic, the recommendations to date are often very broad, without clear guidance for specific settings, although some more specific guidelines are starting to emerge.^8^

One approach to facilitate the lifting of lockdown measures and to limit transmission in areas where lockdowns cannot be imposed would be to implement and monitor specific measures for “precision physical distancing.” Whereas “precision medicine” offers medical care tailored to optimize efficiency or therapeutic benefit for individual patients, precision physical distancing would tailor physical distancing within specific physical, social, cultural, political, and economic contexts and to specific groups and settings. For example, specific precision physical distancing measures could be developed for workplaces, gatherings, and community events such as weddings and funerals, places of worship, educational settings, transport sectors, sporting events, and informal settlements.

The more specific prescriptive measures of precision physical distancing would have numerous advantages. Because they prescribe a specific preventive behavior, they can be more strictly encouraged, monitored, and, if need be corrected or enforced, both on an institutional and individual basis. For example, implementing a set of precision physical distancing practices can be a condition, with a certificate issued, for a restaurant to reopen and remain in business, analogous to passing health inspection (Table 1). Although this enhanced monitoring will require enhanced human and financial resources, these will certainly be less economically damaging than a prolonged lockdown.

**Table 1:** Examples of precision physical distancing guidelines

Restaurant
Seating capacity restricted based on specific assessment of sufficient distancing based on configuration of the establishment
All customers seated until given permission to circulate by management (e.g., to exit or use toilet facilities)
Controlled entry and exit to ensure spacing
Tables must be at least 2 m apart, with tape or chalk lines on the floor to mark out spacing and pathways to exits and toilet facilities
Maximum of two people per table, not seated face-to-face (to avoid droplet deposition on mucous membranes during speaking, coughing or sneezing)
No touching between clients or staff
Hand sanitizer and public health message on precision physical distancing and healthy practices to avoid COVID-19 placed on each table
Servers must wear masks at all times
Tables and chairs are decontaminated between customers
Local food market
Controlled entry and exit to ensure spacing
Entry restricted based on specific assessment of sufficient distancing based on configuration of the market
Every other stall left empty, with monitors to reinforce and control flow (e.g., movement between stalls)
No more than two people at a time per stall
Hand sanitizer or sanitation station with soap and water placed in front of each stall
Surfaces where client–seller interface occurs decontaminated after each client interaction, with complete decontamination of market at the end of each day
Sporting event*
Controlled entry and exit to ensure spacing
Seating capacity restricted based on specific assessment of sufficient distancing based on the configuration of the venue and section of stands
Every other seat left empty, with monitors in each section to reinforce and control movement and flow (e.g., to exit or use toilet facilities)
Hand sanitizer or sanitation station with soap and water placed at entrance to each section of stands
No food or drink sold
Stadium decontaminated after each game

* In some settings, additional measures could be used to safely “unlock,” such as targeted laboratory testing to ensure safety of players engaged in sporting events. For example, a point-of-care assay for COVID-19, for which research and development are rapidly advancing, could be used for a football game to be safely played and televised to a public eager to return to the distractions that they love, after ensuring that all players tested negative via rapid test performed just hours before the game.

In the United Kingdom, physical distancing measures were associated with a 73% reduction in the daily number of contacts observed per participant, with an important projected reduction in transmission, but continued tracking and assessment of the contribution of specific measures will be essential in guiding policies on specific behaviors to keep transmission below levels that sustain the pandemic.^9,10^ In addition to facilitating monitoring and enforcement, the more prescriptive approach of precision physical distancing will facilitate scientific studies to assess the efficacy of specific measures; for example, comparing infection incidence in workplaces where different variants of precision physical distancing have been applied.

An advantage of precision physical distancing relative to many other measures is that it can be developed at a low cost and adapted to diverse sociocultural and economic settings, including those in which the complexity or social cost of maintaining a lockdown is high. Guidelines could be developed collaboratively on a very local level by local leaders, potentially even on a neighborhood scale. In places where COVID-19 has yet to be introduced or where widespread transmission is yet to occur, early implementation of precision physical distancing, along with other measures, could even help avoid the necessity of a lockdown. Importantly, this approach offers the essential component of engaging the community as a central partner in the fight against COVID-19, countering the implicit “us against them” message of government imposition of lockdowns. Village chiefs or elected representatives could hold the equivalent of town hall meetings with local business and religious leaders to identify specific transmission risks related to the common economic activities and social customs in their village or district, and then work with public health experts to devise practical solutions based on precision physical distancing. However, although the advantages of decentralized control are clear, care must be taken to guard against individuals and communities that seek to implement policies that are not evidence based or even contrary to public health advice, with the potential for negative impact, including increased COVID-19 transmission, in their community and beyond. The principle of keeping guidance evidence based, to the extent evidence exists, must always be respected.

In addition to some form of distancing, cardinal features of the response to COVID-19 remain enhanced hygienic practices (e.g., frequent handwashing and avoiding touching the face), extensive testing, case identification and treatment, containment through quarantine (including lockdowns and reverse quarantine (or “shielding”) of vulnerable groups),^4^ and effective risk communication to promote healthy behavior change. Precision physical distancing represents an important tool in this public health armamentarium. All must be adapted to the sociocultural and economic contexts, the available resources of the country and community, and evolution of the epidemic in a given place and time.

An idle, stressed, economically dormant, and beleaguered population in lockdown is not healthy or sustainable for the individual or state. Providing the population hope and a means of participating in solutions is essential. As the COVID-19 pandemic evolves, precision physical distancing is one important component of a sustainable long-term solution that is proportionate to the risk yet does not have a disproportionate impact on society and the economy, allowing a partial return to normal activities, with the community as an essential partner.
